# Development and validation of an inflammatory response-related signature in triple negative breast cancer for predicting prognosis and immunotherapy

**DOI:** 10.3389/fonc.2023.1175000

**Published:** 2023-06-15

**Authors:** Yangyang Guo, Kenan Cen, Shi Yang, Yifeng Mai, Kai Hong

**Affiliations:** ^1^ Department of Thyroid and Breast Surgery, Ningbo First Hospital, Ningbo, Zhejiang, China; ^2^ Department of Geriatrics, The Affiliated Hospital of Medical School of Ningbo University, Ningbo, Zhejiang, China

**Keywords:** inflammatory response, TNBC, immune microenvironment, immunotherapy, prognosis

## Abstract

**Background:**

Inflammation is one of the most important characteristics of tumor tissue. Signatures based on inflammatory response-related genes (IRGs) can predict prognosis and treatment response in a variety of tumors. However, the clear function of IRGs in the triple negative breast cancer (TNBC) still needs to be explored.

**Methods:**

IRGs clusters were discovered via consensus clustering, and the prognostic differentially expressed genes (DEGs) across clusters were utilized to develop a signature using a least absolute shrinkage and selection operator (LASSO). Verification analyses were conducted to show the robustness of the signature. The expression of risk genes was identified by RT-qPCR. Lastly, we formulated a nomogram to improve the clinical efficacy of our predictive tool.

**Results:**

The IRGs signature, comprised of four genes, was developed and was shown to be highly correlated with the prognoses of TNBC patients. In contrast with the performance of the other individual predictors, we discovered that the IRGs signature was remarkably superior. Also, the ImmuneScores were elevated in the low-risk group. The immune cell infiltration showed significant difference between the two groups, as did the expression of immune checkpoints.

**Conclusion:**

The IRGs signature could act as a biomarker and provide a momentous reference for individual therapy of TNBC.

## Introduction

The incidence rate of breast cancer (BC) increases each year among women, making it the most common malignant tumor for women (1). According to the expression of hormone receptors, we call a type of breast cancer that lacks the expression of estrogen receptor (ER), progesterone receptor (PR) and human epithelial growth factor receptor 2 (Her-2) as triple negative breast cancer (TNBC) (2, 3). TNBC is a special type of BC, accounting for 10%~20% of the disease (4). TNBC does not express hormone receptors and Her-2, cannot benefit from endocrine therapy and Her-2 targeted therapy, and their pathological characteristics, treatment and prognosis are very different from those of other types of breast cancer, which has attracted much attention (5). Due to the lack of effective treatment, the prognosis of TNBC is very poor (6). Therefore, it is urgent to develop a model to evaluate the prognosis and provide personalized treatment for TNBC patients. In addition, a comprehensive analysis rather than a single factor is necessary to find reliable prognostic biomarkers that can help guide the treatment strategy of patients with TNBC.

Inflammation is one of the most important characteristics of tumor tissue (7). Many physical environmental factors, including dietary factors, carcinogenic microorganisms, pollutants, tobacco smoke and particulate matter, can cause chronic inflammation of multiple organs and systems (8, 9). Without intervention, chronic inflammatory reaction may lead to the occurrence of tumor (10). Signatures based on inflammatory response-related genes (IRGs) correlate to prognosis and treatment response in a variety of tumors, including bladder cancer (11), pancreatic tumor (12), esophageal cancer (13) and hepatocellular carcinoma (14). However, the role of inflammation related genes in TNBC remains unclear.

As mentioned above, targeting biomarkers related to inflammatory response may be a promising new option for tumor treatment. A large number of inflammation-related regulatory factors are related to the progression of TNBC (15). However, TNBC is a disease caused by multiple genes and pathways (16). Considering the limitations of a single biomarker, we screened the prognostic relevance of multiple IRGs and constructed a novel signature for risk stratification and prognostic evaluation of patients. Here, our goal is to establish an inflammatory related prognosis model to predict the outcome of TNBC. We used TCGA and GEO databases to develop and validate the prognostic characteristics based on IRGs, which can evaluate the prognosis and treatment response of patients with TNBC.

## Materials and methods

### Data obtain

The TCGA database (https://portal.gdc.cancer.gov) was searched to obtain the gene expression data (measured in fragments per kilobase million, or FPKM) of 160 TNBC tumor samples, 111 normal tissue samples, and the related clinical data. TCGA-TNBC was randomly categorized into the train and test groups according to the 1:1 ratio with R software (**Supplementary Table S1**). The GSE21653 and GSE58812 were downloaded from GEO database (https://www.ncbi.nlm.nih.gov/geo/) (**Supplementary Files S1**, **S2**). A search of the MSigDB database (http://www.broad.mit.edu/gsea/msigdb/) yielded 200 IRGs. We identified the gene set from the MSigDB database by inputting the Keywords “inflammatory” and selecting the Filters “hallmark gene set + homo sapiens” in the “Search Human Gene Sets” section, and the genes are listed in **Supplementary Table S2**.

### Consensus clustering analysis

The R package “limma” and “ConsensusClusterPlus” were used for consistent cluster classification of TNBC. The filter of |log fold change (FC)| was set as 1 and the filter of FDR was set as 0.05 (17). The association between clusters and overall survival (OS) was analyzed by R packet “survival” (18, 19). The results were analyzed by R packages “pheatmap”, “survival” and “survminer” as heat map and Kaplan-Meier (KM) curves (20). The “limma” program was employed to determine the differentially expressed genes (DEGs) between two clusters with the criteria of logFC >1 and FDR < 0.05. Scores of infiltrating immune cells were derived via the MCPcounter method, and the difference in infiltration between the two subtypes was assessed, and P < 0.05 was considered as significant (21).

### Development and verification of the prognostic signature

Prognostic DEGs were determined by univariate Cox regression analysis, and P < 0.05 was considered to be significant. A prognostic signature was then derived by integrating four genes based on multivariate Cox regression and least absolute shrinkage and selection operator (LASSO) analyses (22). The median risk score was used to classify individuals with TNBC into two categories (low- and high-risk categories). Subsequently, the OS was compared by KM analysis, and P < 0.05 was considered as significant. OS and Receiver Operating Characteristics (ROC) of subgroups were analyzed with the “survival”, “survminer” and “timeROC” R packages for 1, 3, and 5 years (23). Specifically, the “ggplot2” R program was employed to conduct a principal component analysis (PCA) (24). By incorporating risk assessment with clinical data, a nomogram was developed. Next, multifactor ROC was implemented to verify the predictive accuracy of the nomogram.

### Comparative analysis of the tumor microenvironment

Immune cell abundance (ImmuneScores) was calculated by the ESTIMATE (25). To examine the variation in diverse immune cells between two categories, we used the TIMER, CIBERSORT-ABS, QUANTISEQ, EPIC, MCPCOUNTER, and CIBERSORT, XCELL, algorithms (26). Differential immune microenvironment was probed via single-sample gene set enrichment analysis (ssGSEA) (27). The expression of immune-related genes was also determined, and P < 0.05 was considered as significant. Additionally, we also analyzed the response of two subgroup TNBC to immunotherapy.

### Functional enrichment analysis

Putative cellular functions of DEGs were identified via the Gene Ontology (GO) analysis (28). Besides, underlying pathways related to DEGs were determined by Kyoto Encyclopedia of Genes and Genomes (KEGG) enrichment analysis, and P < 0.05 was considered as significant (29). To assess the probable biological functioning differences between high- and low-risk categories, a gene set variation analysis (GSVA) was carried out, and P < 0.05 was considered as significant (30).

### Drug sensitivity analysis

We investigated the potential for the signature to serve as a predictor for medications used in chemotherapy and targeted treatment. Subsequently, the half-maximal inhibitory concentration (IC50) was computed with the pRRophetic method, and P < 0.05 was considered as significant (31). All the raw code was added in the **Supplementary File S3**.

### RT-qPCR

The tumor cell MDA-MB-231 and normal cell MCF-10A were obtained from the Cell Bank of the Shanghai Institute of Biochemistry and Cell Biology (Shanghai, China). All cells were cultured in DMEM (Gibco), adding 10% FBS (Gibco), 1% penicillin-streptomycin. Trizol was employed to isolate total RNA, after which it was reverse-transcribed into the cDNA template. Next, RT-qPCR was conducted with SYBR Green Real-Time PCR Master Mix Plus (Toyobo). The internal reference gene utilized was β-Actin. The PCR sequence was added in the **Supplementary File S4**.

## Results

### Identification of IRGs clusters in TNBC

**Figure 1:**
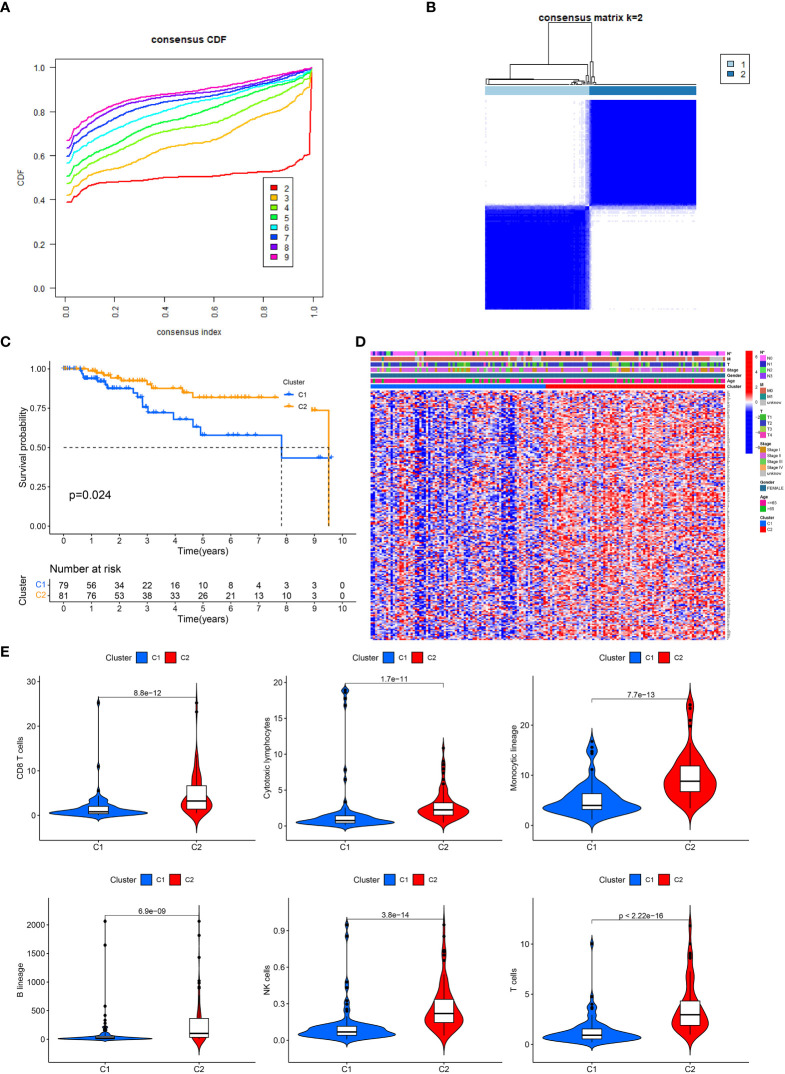
IRGs clusters and clinical characteristics between TNBC samples in two clusters. **(A)** The cumulative distribution function curve illustrates the most effective way of IRGs clustering. **(B)** The consensus matrix of the clustering analysis via k-means clustering (k = 2). **(C)** Kaplan–Meier (KM) curves for the overall survival (OS) of TNBC patients among different IRGs groups. **(D)** Heatmap of IRGs expression in TNBC patients with different clinical characteristics and IRGs clusters. **(E)** The differences in immune cell infiltration between two clusters.

The link between IRGs expression and TNBC subtypes was first analyzed using a consensus clustering method. As depicted in **Figures 1A, B**, the CDF curve was applied to categorize patients with TNBC into two clusters (C1 and C2). In contrast with C2, C1 individuals diagnosed with TNBC had remarkably lower survival duration (**Figure 1C**). The correlation between IRGs clusters, clinical characteristics, and IRGs expression in TNBC patients was depicted in **Figure 1D**. The heatmap showed that the C1 and C2 TNBC had distinct IRGs expression pattern, and the cluster was significantly related to the N stage.

Since immune cells perform an instrumental function in the onset and advancement of TNBC, we next evaluated the variations in infiltrating immune cells between the two clusters. In cluster 1, the level of CD8 T cells, monocytic, cytotoxic lymphocytes, B cells, NK cells and T cells were lower than in cluster 2, which might partly explain the poor prognosis of C1 (**Figure 1E**).

### Development of the IRGs prognostic signature

**Figure 2:**
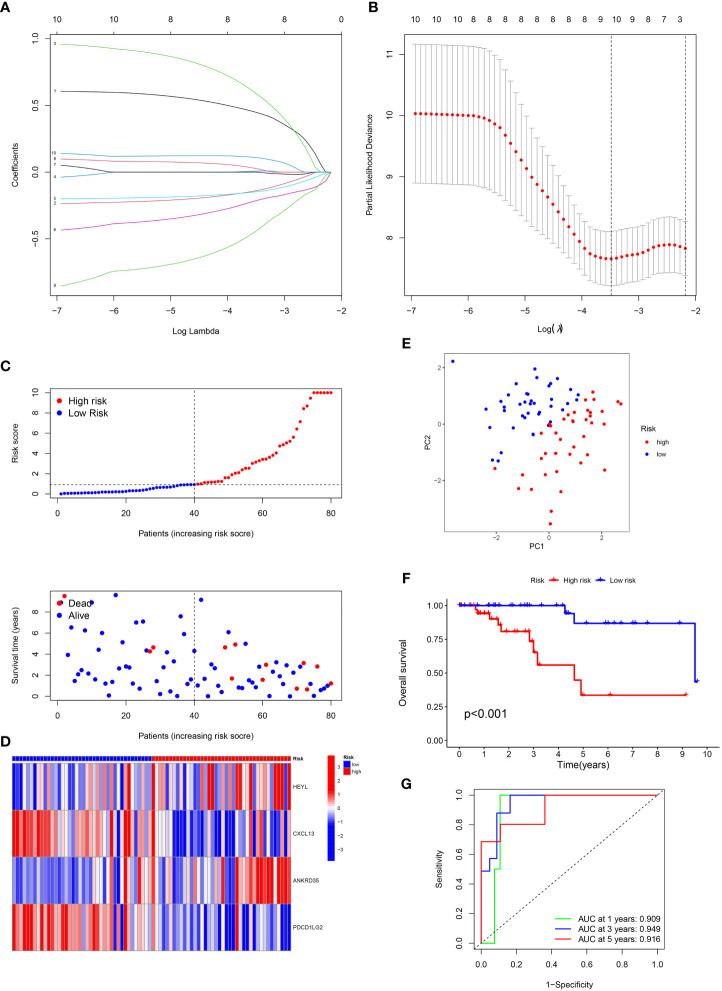
Construction of the prognostic signature. **(A)** LASSO coefficient profiles (y-axis) of the gene sets and the optimal penalization coefficient via 3-fold cross-validation based on partial likelihood deviance. **(B)** The dotted vertical lines represent the optimal values of l. The top x-axis has the numbers of gene sets, whereas the lower x-axis revealed the log (λ). **(C)** Risk score and survival outcome of each case. **(D)** Heatmap showed the expression of risk genes in two risk groups. **(E)** PCA. **(F)** The KM curve showed that patients in the high-risk group had a worse prognosis. **(G)** The AUC for 1-, 3- and 5-years survival.

Using the “limma” program, DEGs were found between two clusters with the criteria of |log fold change (FC)| >1 and FDR < 0.05. Next, 10 prognosis-related DEGs were found by the univariate Cox analysis. Subsequently, we completed a LASSO analysis to remove the overfitting genes and the IRGs signature of four genes (HEYL, CXCL13, ANKRD35 and PDCD1LG2) was created (**Figures 2A, B**). The equation applied to derive the risk score is as indicated: risk score= (HEYL × (0.891821684945936) + (CXCL13 × (-0.322533080452241) + (ANKRD35 × (0.955719797833462) + (PDCD1LG2 × (-1.08495430822516).

Patients with TNBC were classified into low- and high-risk categories according to the median risk score (**Figure 2C**). The variations in the expression of these four genes between the two risk categories are illustrated in **Figure 2D**. PCA analysis showed the perfect separation of high- and low-risk TNBC (**Figure 2E**). Also, patients having high risk scores had a greater fatality rate (**Figure 2F**). Moreover, the ROC curve was performed to assess the IRGs signature, whose AUC values for 1-, 3- and 5-year were 0.909, 0.949, and 0.916, respectively (**Figure 2G**).

### Validation of the IRGs signature

**Figure 3:**
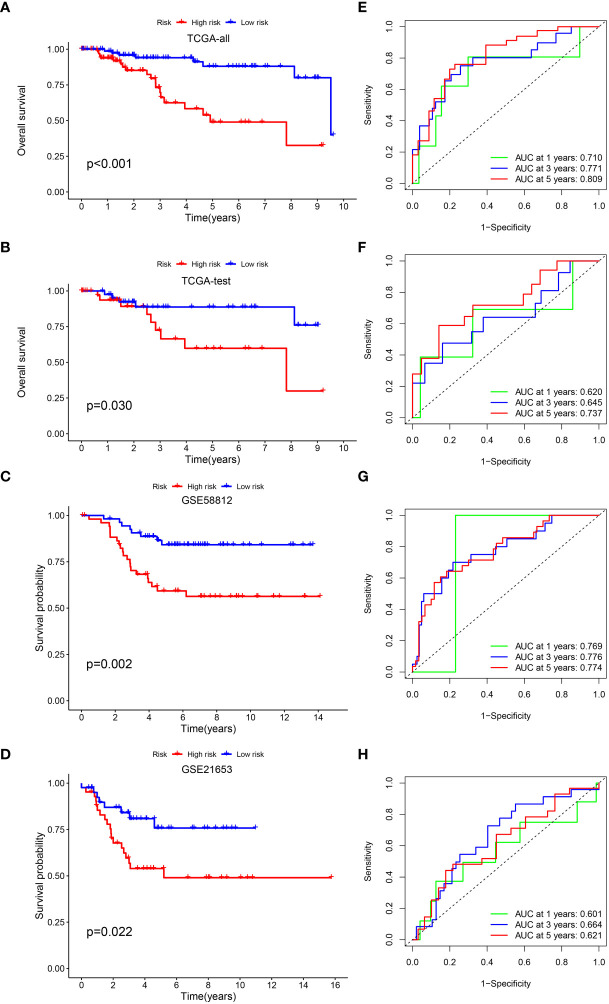
Validation of the prognostic signature. KM curve showed that patients in the high-risk group had a worse prognosis in TCGA-all **(A)**, TCGA-test **(B)**, GSE58812 **(C)** and GSE21653 **(D)**. The AUC for 1-, 3- and 5-years survival in TCGA-all **(E)**, TCGA-test **(F)**, GSE58812 **(G)** and GSE21653 **(H)**.

we verified the aforementioned findings in test datasets. All patients with TNBC in the test datasets were also divided into low- and high-risk categories. The K-M survival curve disclosed that the low-risk individuals exhibited a more favorable prognosis in contrast to those at high risk in TCGA-all, TCGA-test, GSE58812 and GSE21653 (**Figures 3A–D**). The AUC of 1-, 3-, and 5-year periods were 0.710, 0.771, and 0.809, correspondingly, in TCGA-all (**Figure 3E**), 0.620, 0.645, and 0.737 in TCGA-test (**Figure 3F**), 0.769, 0.776, and 0.774 in GSE58812 (**Figure 3G**), and 0.601, 0.664, and 0.621 in GSE21653 (**Figure 3H**). Furthermore, we performed subgroup analysis of survival for single clinical characteristic, finding that low-risk TNBC had significantly better prognosis than high-risk TNBC in age <= 65, T1 + 2, M0, N0, N1 + 3, Stage I+II (**Supplementary Figures S1A–F**). Next, we also compared with the prognosis model of others, and the C-index of our signature was higher than others (**Supplementary Figure S1G**). In addition, RT-qPCR was used to verify the expression of risk genes. Higher expression of CXCL13 and HEYL in tumor cell than normal cell was found, as well as the lower expression of ANKRD35 and PDCD1LG2 (**Supplementary Figure S2**).

### Construction of a nomogram for TNBC

Multivariate and univariate Cox regression analyses proved that risk score independently acted as a robust prognostic marker (P < 0.05) (**Supplementary Figure S3A, B**). An innovative nomogram was developed using the IRGs signature and clinical variables from the TCGA dataset to further exploit the IRGs signature’s prognostic potential (**Supplementary Figure S3C**). Following that, we portrayed the calibration plots in 1, 3, and 5 years, and the calibration curve performed well (**Supplementary Figure S3D**). Additionally, a ROC analysis was conducted to assess the nomogram’s prognosis-predicting value in comparison to other single variables (stage, N, M, T and age). For 1-year survival, the AUCs of nomogram and risk score were 0.887 and 0.696 (**Supplementary Figure S3E**). For 3-year survival, the AUCs of nomogram and risk score were 0.923 and 0.768 (**Supplementary Figure S3F**). For 5-year survival, the AUCs of nomogram and risk score were 0.892 and 0.804 (**Supplementary Figure S3G**). This novel nomogram proved to be an excellent model for prognosis prediction.

### The TME analysis for high- and low-risk TNBC

**Figure 4:**
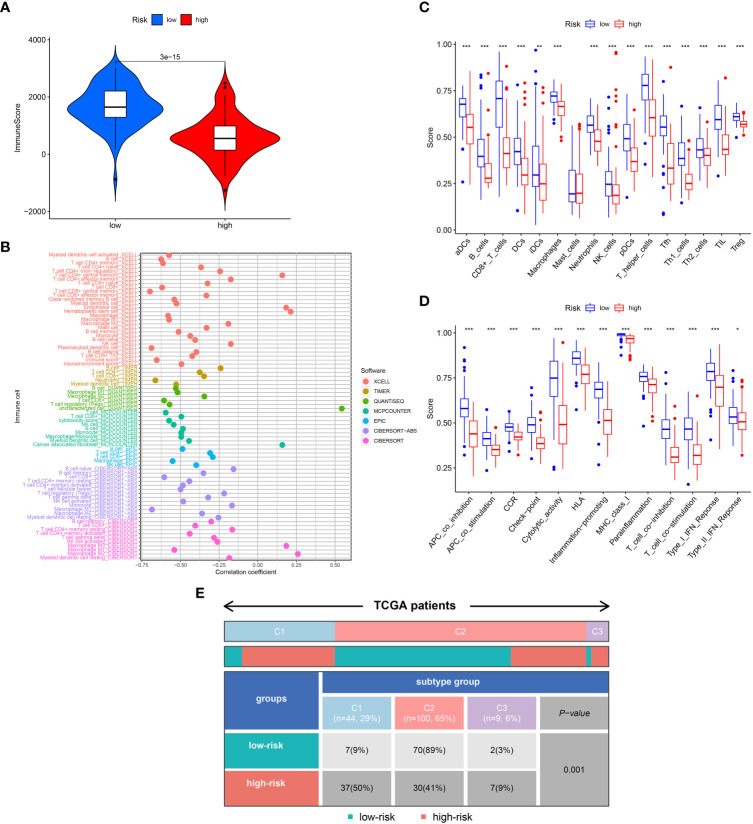
Analysis of immune conditions of high- and low-risk groups. **(A)** Differences in immune score between the two groups. **(B)** The analysis of differences in immune cell infiltration between the two groups with Multiple algorithms. **(C)** The analysis of differences in immune cell infiltration between the two groups with ssGSEA. **(D)** The analysis of differences in immune functions between the two groups with ssGSEA. **(E)** The distribution of patients with high- and low-risk in different immune subtypes. "*" represented P <0.05, "**" represented P <0.01, and "***" represented P <0.001.

Biological behavior of the tumor can be determined by the TME. ESTIMATE analysis revealed that the ImuneScores lower in the high-risk category in contrast with the low-risk category (**Figure 4A**). The distinctions of immune cell infiltration were also explored via CIBERSORT, MCPCOUNTER, QUANTISEQ, EPIC, TIMER, CIBERSORT-ABS, and XCELL. As shown in **Figure 4B**, the low-risk category had remarkably higher levels in most immune cells. In addition, ssGSEA analysis found less infiltration of the B cells, CD8+ T cells, tumor-infiltrating cell (TIL), Neutrophils, T helper cells, and T cells regulatory (Treg) in the high-risk patients in contrast with the low-risk patients (**Figure 4C**). Most immunologic functions, including T cells co-stimulation, CCR, Type II IFN response, and T cell co-inhibition were also improved in the low-risk patients (**Figure 4D**). This may explain why the low-risk category has a superior prognosis. Additionally, **Figure 4E** depicted the distribution of low- and high-risk individuals across multiple immune subtypes.

**Figure 5:**
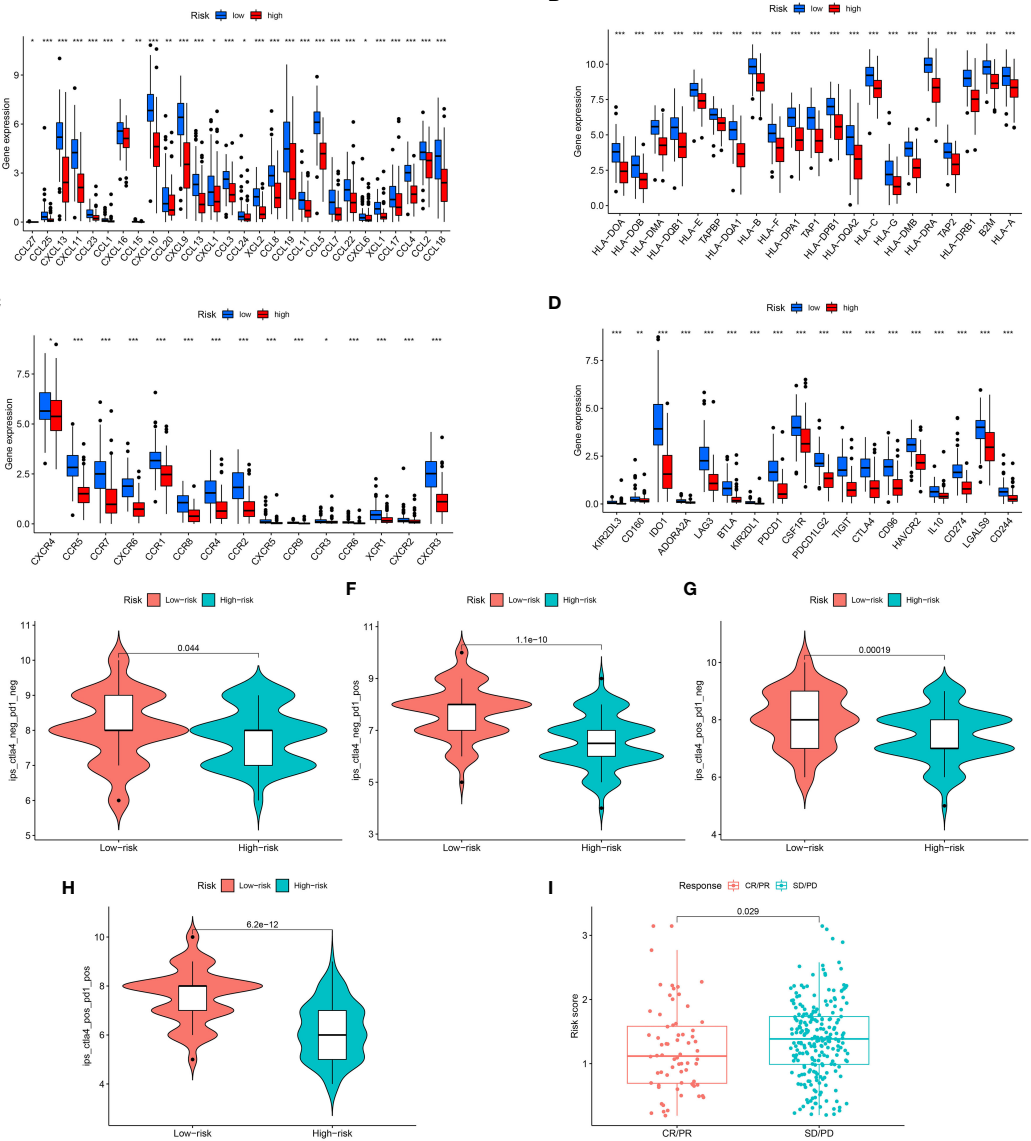
Assessment of Immunotherapy response of high- and low-risk groups. **(A–D)** The immune-related gene expression levels in different groups. **(E–H)** Violin plots showed the relationship between IPSs and risk groups. **(I)** Prediction of immunotherapy response. "*" represented P <0.05, "**" represented P <0.01, and "***" represented P <0.001.

We next examined the low- and high-risk patients in terms of the expression patterns of immune-related genes. A majority of immune-related genes were discovered to be expressed at low levels in the high-risk category (**Figures 5A–D**). The public dataset TCIA (The Cancer Immunome Atlas, https://tcia.at/home) was then used to estimate the responsiveness of CTLA-4 and PD-1 immune checkpoint inhibitors based on the above results. A striking finding of the analysis was that low-risk patients responded more strongly to anti-CTLA-4 and anti-PD-1 treatments as compared with high-risk patients (**Figures 5E–H**). Furthermore, low-risk patients respond better to immunotherapy in contrast with those at high-risk in immunotherapy dataset (**Figure 5I**).

### Functional enrichment of the IRGs signature

**Figure 6:**
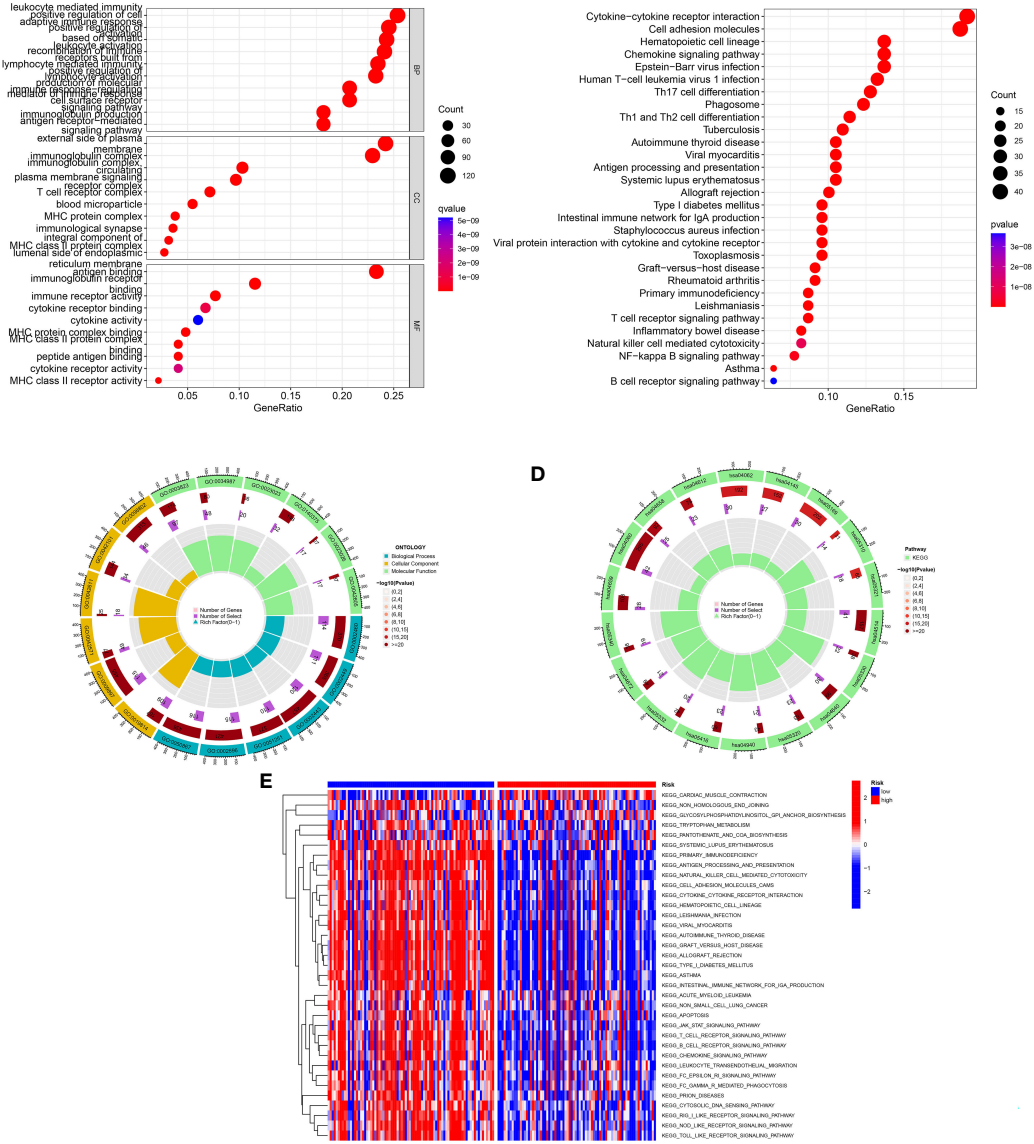
Function analysis. **(A, B)** GO analysis of differential genes between high and low-risk groups. **(C, D)** KEGG analysis of differential genes between high- and low-risk groups. **(E)** GSVA enrichment analysis in high- and low-risk groups.

GO and KEGG enrichment analyses were conducted to investigate the latent biological roles of the IRGs signature. The GO result revealed that the DEGs between low- and high-risk TNBC were primarily enriched in lymphocyte mediated immunity, immunoglobulin complex and immune receptor activity (**Figures 6A, B**). The KEGG result suggested that the DEGs were primarily enriched in immune-related signaling pathway, including cytokine-cytokine receptor interaction, chemokine signaling pathway, and Th1 and Th2 cell differentiation (**Figures 6C, D**). Moreover, GSVA results showed substantial differences of signaling pathways between patients with high- and low-risk TNBC (**Figure 6E**).

### Drug sensitivity analysis

**Figure 7:**
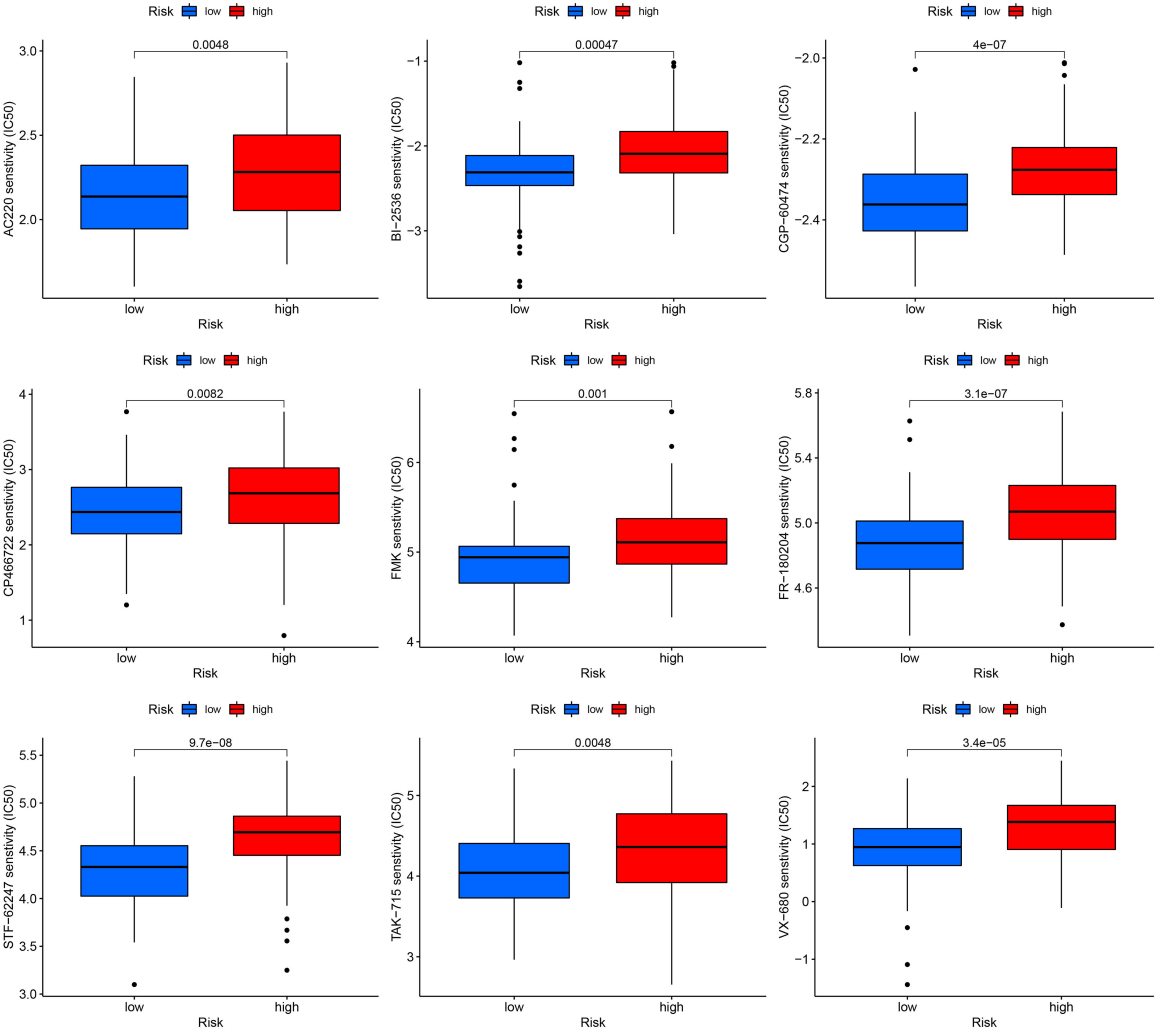
Drug sensitivity analysis in high and low-risk groups.

We correlated the TNBC patients’ risk scores with the IC50 values of chemotherapy and targeted treatment medications to learn more about the possible variations in drug sensitivity between low- and high-risk categories. The IC50 values of 9 drugs (AC220, BI-2536, CGP-60474, CP466722, FMK, FR-180204, STF-62247, TAK-715 and VX-680) were significantly higher in high-risk group, indicated that low-risk TNBC were more sensitive to the drugs (**Figure 7**).

## Discussion

TNBC is a subtype with the worst prognosis in breast cancer, and visceral metastasis occurs at the early stage of the disease. The recurrence rate is high after surgical resection, the lack of molecular targeted drug, the poor effect of endocrine therapy, the different effects of postoperative chemotherapy, and the short survival period of TNBC patients need to find a new treatment (32). Tumor occurrence, development, and metastasis are associated with inflammation (33, 34). Research has confirmed that when the number of neutrophils and monocytes in peripheral blood is increased and the number of lymphocytes and monocytes is reduced, cancer is more prone to progression and recurrence (35). In this study, 10 IRGs related to prognosis of TNBC were screened by mining TCGA and GEO databases. The 10 screened IRGs were analyzed by LASSO to construct a prognosis model for TNBC. Multivariate Cox regression analysis confirmed the ability of the risk score to predict TNBC outcome independently. In both the train set and validation set, survival rates differed between high-risk and low-risk groups.

In this study, the 4 genes that constructed the prognosis signatre for TNBC were HEYL, CXCL13, ANKRD35 and PDCD1LG2. HEYL is a downstream gene of the Notch and transforming growth factor-β pathways. Kuo et al. found that HEYL might be a tumor suppressor of liver carcinogenesis by activating P53-mediated apoptosis and up-regulating P53 gene expression (36). *In vivo*, HEYL modulates metastasis-forming capacity of spheroid cells derived from colorectal cancer patients (37). As a chemokine derived from a B-cell motif, CXCL13 plays an important role in the immune system (38). Blocking CXCL13 promotes apoptosis in MDA-MB-231 cells, inhibiting their proliferation. This effect may be related to the down-regulation of CXCL13 and the inhibition of CXCR5/ERK signaling pathway (39). Dai et al. Found that CXCL13 and its receptor CXCR5 were significantly correlated in ccRCC tissues. The prognosis of ccRCC patients with high CXCL13 and high CXCR5 expression was the worst. By binding to CXCR5 and activating the PI3K/AKT/mTOR signal pathway, CXCL13 promoted proliferation and migration of ccRCC cells (40). In colorectal cancer patients, PDCD1LG2 expression is negatively correlated with Crohn’s-like lymphoid reactions, suggesting a possible link between PDCD1LG2-expressing tumor cells and adaptive antitumor immunity (41). The increased expression of PDCD1LG2 in pancreatic cancer is related to higher tumor grade, poorer prognosis, higher clinical stage, and worse molecular subtype and FAK promotes immune escape of pancreatic cancer through regulating PDCD1LG2 (42). However, the role of these genes in TNBC remains to be further explored.

The tumor microenvironment (TME) is constituted of diverse immune cells, interstitial cells, extracellular matrix, and tumor blood vessels, which stimulate the onset and advancement of cancer. During tumor progression and tumorigenesis, immune cells infiltrate TME at varying levels (43). Our analysis illustrated that TNBC patients having high risk scores recorded lower ImuneScores. We found most of the immune cells (B cells, CD8+ T cells, Treg, T helper cells, Neutrophils and TIL) were substantially reduced in the high-risk patients in contrast with the low-risk patients. Additionally, the majority of immune-related genes tended to be downregulated in the high-risk population, whereas the low-risk category illustrated considerable improvement in immunologic function. Research suggests that immune cells are important components of anti-tumor immunity (44). One reason high-risk individuals have such a dismal prognosis is that they have fewer immune cells and attenuated immunological functioning. Results highlighted that low-risk individuals with TNBC responded more positively to immunotherapy compared to those in the high-risk category. The findings of this research shed light on the involvement of IRGs in TNBC and may be utilized to direct immunotherapeutic and chemotherapeutic interventions for TNBC patients.

In addition, this study also explored the differences in biological processes, signal pathways and immune functions between high-risk and low-risk groups. Through GO and KEGG enrichment analysis, the DEGs between high-risk and low-risk patients were analyzed, and the results showed that these genes were associated with immune-related signaling pathway, including lymphocyte mediated immunity, immune receptor activity cytokine-cytokine receptor interaction, Th1 and Th2 cell differentiation and chemokine signaling pathway. The analysis of immune cell and immune function further suggest that there is significant difference in immune cell and immune function between high-risk group and low-risk group. These results suggest that IRGs may participate in the regulation of the prognosis of TNBC by regulating immunity.

Nevertheless, our investigation does have a few drawbacks. Case selection bias could be present since the vast majority of analyses use data from publicly available data sets and all samples are retrieved retroactively. Second, The AUC value of the signature in the GSE21653 dataset was lower than 0.65, which might be due to the high heterogeneity of TNBC. TNBC was an invasive breast cancer type with variable genome; however, our signature is applicable to whole TNBC, limiting the lack of more detailed raw data. This problem can be solved using more detailed data in the future. Furthermore, the prediction of immunotherapy is based on some novel biomarkers such as ImmuneScore, IPS, and immunotherapy response via IMvigor210 cohort data. We lack the real-world data of immunotherapy response data for TNBC. Therefore, our next plan is to conduct relevant clinical research. Finally, additional *in vitro* and *in vivo* tests are warranted to corroborate our findings.

In summary, we designed a molecular cluster and prognostic signature based on IRGs, which aid in anticipating survival, directing immunotherapy, and determining clinical outcomes. This research potentially provides deeper insights into the function of IRGs in TNBC and facilitates the development of more effective therapies for this disease.

## Data availability statement

The original contributions presented in the study are included in the article/**Supplementary Material**. Further inquiries can be directed to the corresponding authors.

## Author contributions

YG and KH designed the study and drafted the manuscript. KC, SY and YM wrote the manuscript. KH and KC searched the relevant data. SY and YG analyzed the original data. All authors contributed to the article and approved the submitted version.

## Supplementary material

Supplementary Table 1 Clinical information of TNBC in TCGA-all, TCGA-train and TCGA-test datasets.

Supplementary Table 2 The inflammatory response-related genes.

Supplementary Figure 1 Survival curve after grouping according to clinical features, including **(A–F)** age ≤ 65, T1 + 2, M0, N0, N1-3, Stage I+II. **(G)** Comparison between our model and others’ model.

Supplementary Figure 2 RT-qPCR of HEYL, CXCL13, ANKRD35 and PDCD1LG2 in tumor and normal cells with three replications.

Supplementary Figure 3 Construction and assessment of nomogram. The forest plot for univariate Cox **(A)** and multivariate Cox regression **(B)** considering clinical indicators and IRGs-score in TCGA cohort. **(C)** The prediction of nomogram in the TCGA dataset. **(D)** Calibration plots for the nomogram. The multifactor AUC for 1- **(E)**, 3- **(F)**, and 5-years **(G)** survival.

Supplementary File S1 Clinical information of GSE21653.

Supplementary File S2 Clinical information of GSE58812.

Supplementary File S3 Raw code of the whole analysis.

Supplementary File S4 PCR sequence of the genes.
