# Supplementary figures and images for: Development and validation of an inflammatory response-related signature in triple negative breast cancer for predicting prognosis and immunotherapy
Source: Front Oncol. 2023 Jun 15;13:1175000. doi: 10.3389/fonc.2023.1175000 (PMC10311032; doi:10.3389/fonc.2023.1175000)

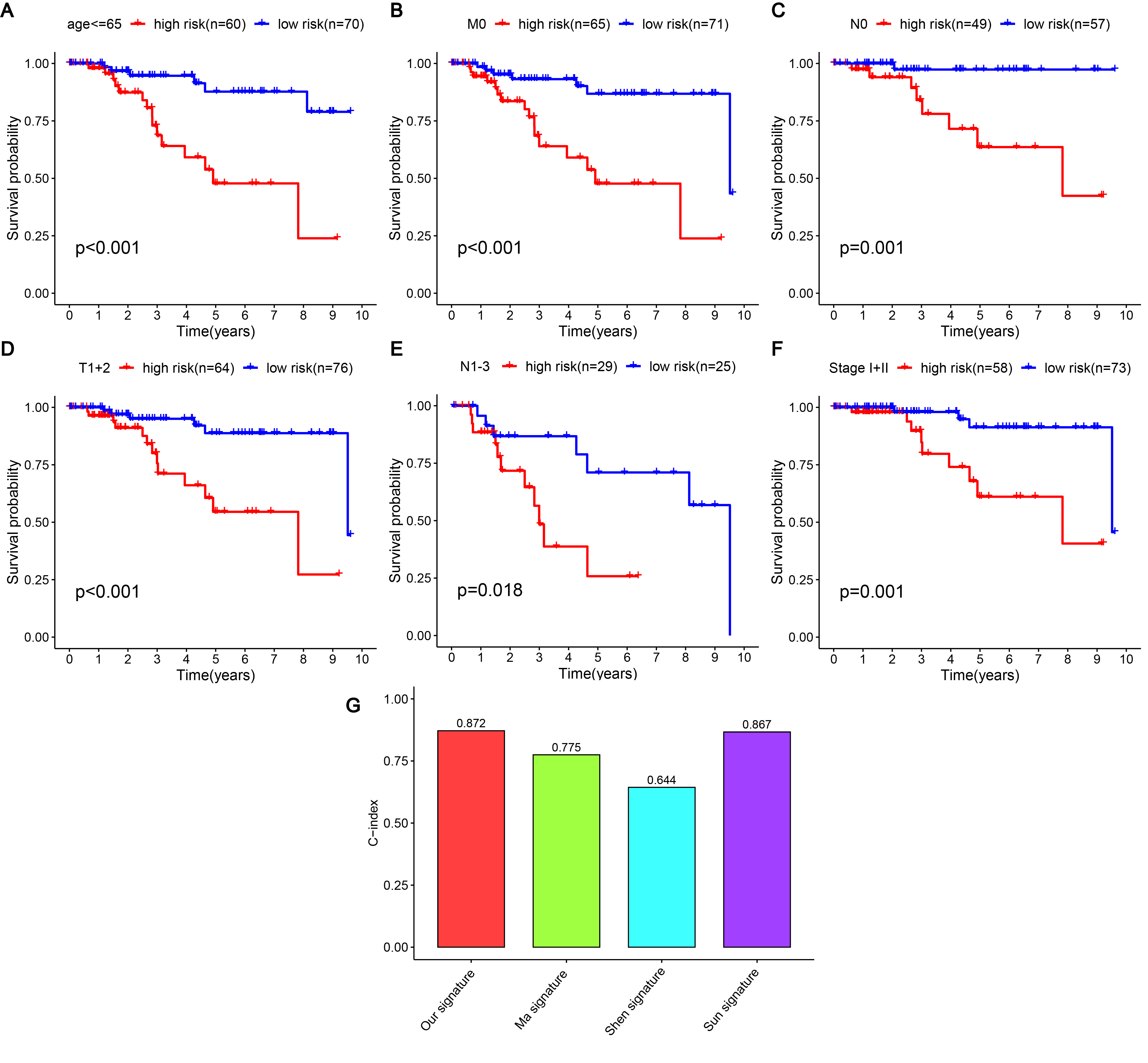

Supplement: Supplementary Figure 1 — Survival curve after grouping according to clinical features, including (A–F) age ≤ 65, T1 + 2, M0, N0, N1-3, Stage I+II. (G) Comparison between our model and others’ model. [file Image_1.jpeg]

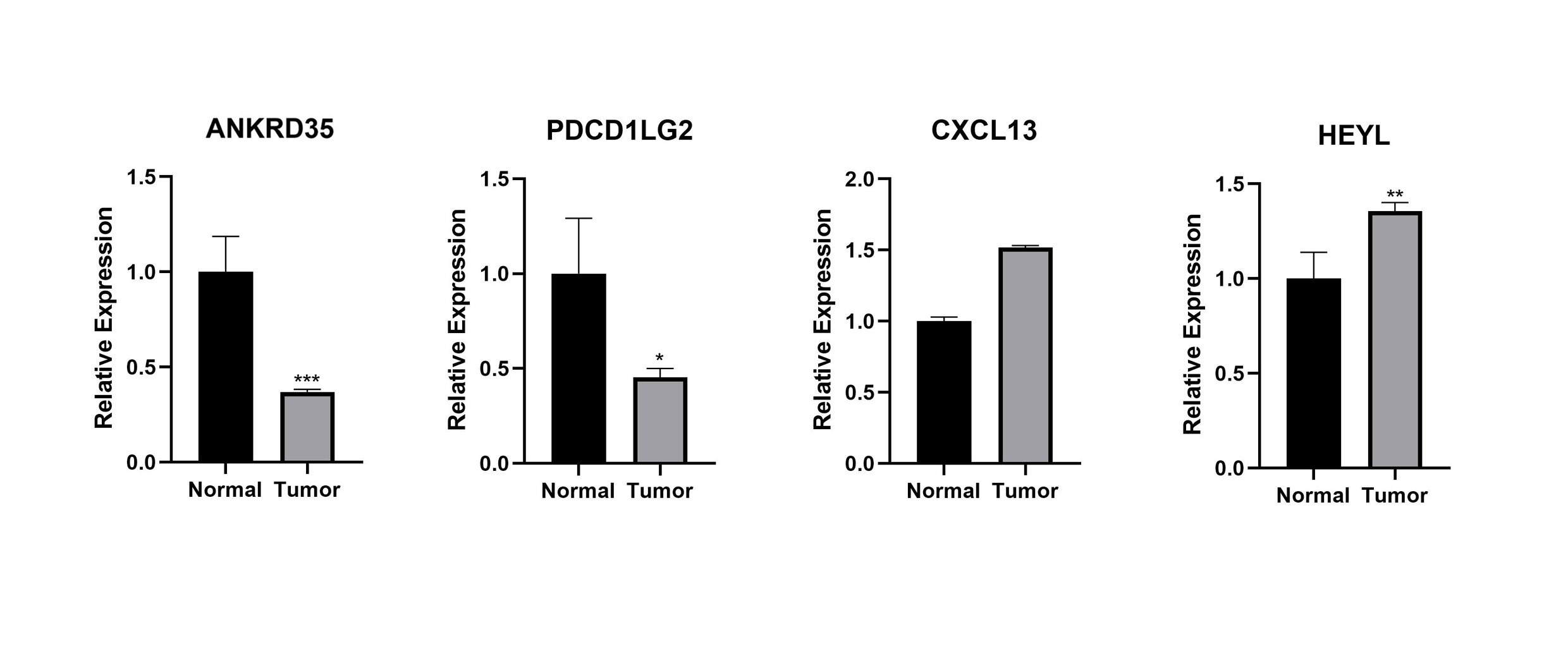

Supplement: Supplementary Figure 2 — RT-qPCR of HEYL, CXCL13, ANKRD35 and PDCD1LG2 in tumor and normal cells with three replications. [file Image_2.jpeg]

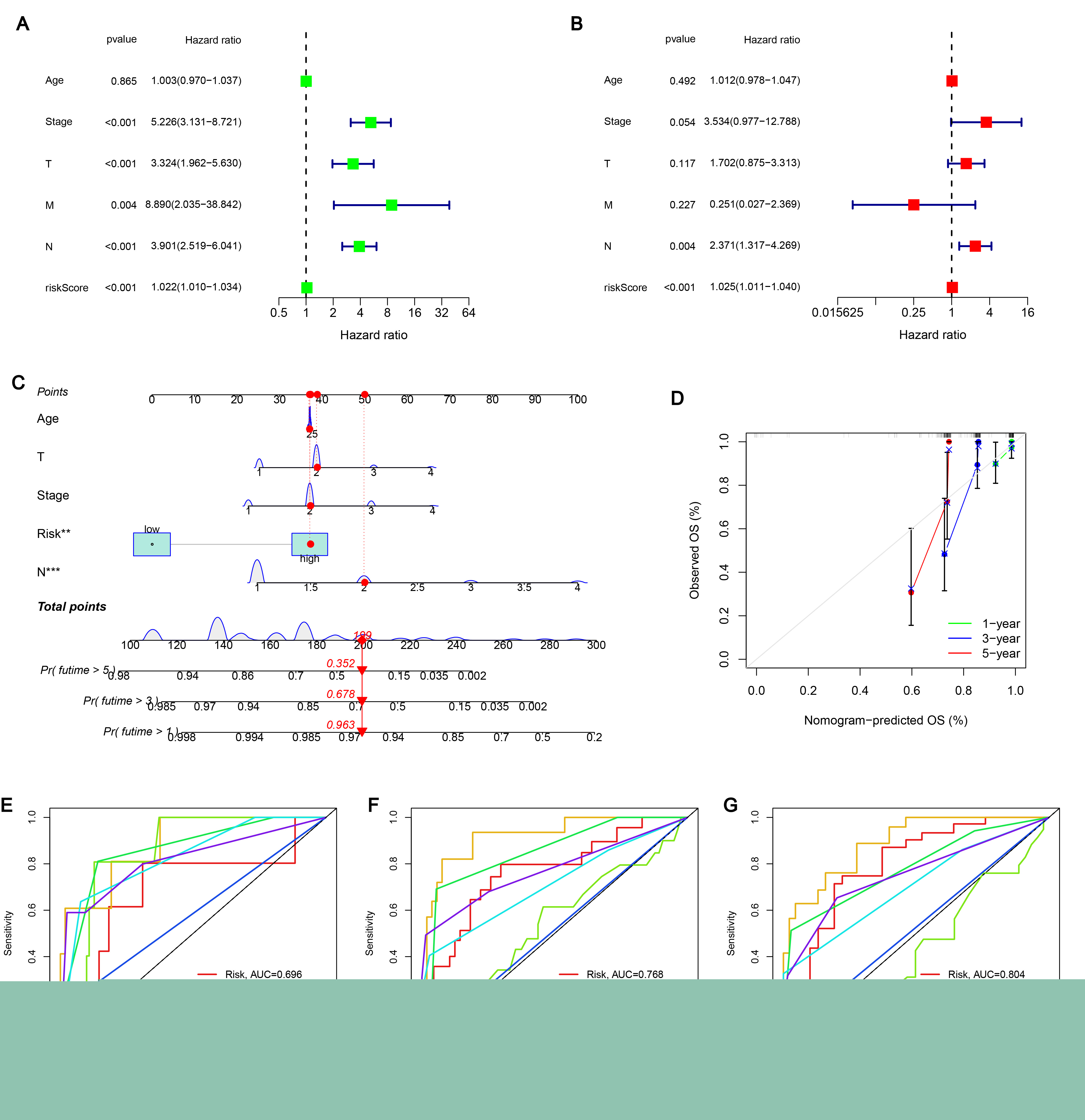

Supplement: Supplementary Figure 3 — Construction and assessment of nomogram. The forest plot for univariate Cox (A) and multivariate Cox regression (B) considering clinical indicators and IRGs-score in TCGA cohort. (C) The prediction of nomogram in the TCGA dataset. (D) Calibration plots for the nomogram. The multifactor AUC for 1- (E), 3- (F), and 5-years (G) survival. [file Image_3.jpeg]
