# Supplementary material for: Development and validation of an inflammatory response-related signature in triple negative breast cancer for predicting prognosis and immunotherapy
Source: Front Oncol. 2023 Jun 15;13:1175000. doi: 10.3389/fonc.2023.1175000 (PMC10311032; doi:10.3389/fonc.2023.1175000)
Supplement: Supplementary File S1 — Clinical information of GSE21653. [file DataSheet_1.docx]

id futime fustat

GSM540113 0.824166667 1

GSM540115 15.75666667 0

GSM540117 3.036666667 1

GSM540118 8.835 0

GSM540119 0.988333333 1

GSM540123 0 1

GSM540125 3.274166667 0

GSM540127 1.818333333 1

GSM540130 9.714166667 0

GSM540132 3.060833333 0

GSM540138 0.936666667 1

GSM540140 0.911666667 1

GSM540143 6.721666667 0

GSM540144 8.09 0

GSM540146 2.669166667 1

GSM540151 7.879166667 0

GSM540157 3.08 1

GSM540158 10.48083333 0

GSM540159 10.78416667 0

GSM540160 10.97583333 0

GSM540164 10.045 0

GSM540167 4.6 1

GSM540176 1.864166667 1

GSM540178 3.54 0

GSM540179 7.094166667 0

GSM540181 9.480833333 0

GSM540183 0.766666667 1

GSM540195 9.160833333 0

GSM540201 2.004166667 1

GSM540209 9.166666667 0

GSM540214 5.190833333 0

GSM540215 8.46 0

GSM540219 8.941666667 0

GSM540237 5.065 0

GSM540238 1.454166667 1

GSM540242 4.14 0

GSM540248 4.076666667 0

GSM540249 7.950833333 0

GSM540250 8.013333333 0

GSM540254 3.756666667 0

GSM540255 0.4575 0

GSM540256 5.656666667 0

GSM540260 6.998333333 0

GSM540261 5.169166667 0

GSM540263 4.8625 0

GSM540264 3.929166667 0

GSM540271 5.514166667 0

GSM540273 0 1

GSM540276 1.116666667 1

GSM540281 4.3725 0

GSM540285 4.7775 0

GSM540292 2.979166667 0

GSM540293 2.803333333 1

GSM540294 2.365833333 0

GSM540302 1.616666667 1

GSM540339 2.5025 1

GSM540345 2.535 1

GSM540346 1.880833333 1

GSM540348 7.764166667 0

GSM540349 1.475833333 1

GSM540350 0.279166667 1

GSM540351 3.003333333 1

GSM540352 5.196666667 1

GSM540353 4.1175 0

GSM540354 4.558333333 0

GSM540355 1.171666667 0

GSM540356 4.539166667 0

GSM540357 4.596666667 0

GSM540358 3.485 0

GSM540359 2.935 0

GSM540360 3.126666667 0

GSM540361 3.145833333 0

GSM540362 2.970833333 0

GSM540363 2.565 0

GSM540364 2.19 0

GSM540365 2.261666667 0

GSM540366 1.169166667 1

GSM540367 1.9025 0

GSM540368 0.615833333 0

GSM540369 0.675833333 0

GSM540370 0.645833333 0

GSM540371 1 1

GSM540372 0.955833333 0

GSM540373 0.640833333 0
