# Supplementary material for: Development and validation of an inflammatory response-related signature in triple negative breast cancer for predicting prognosis and immunotherapy
Source: Front Oncol. 2023 Jun 15;13:1175000. doi: 10.3389/fonc.2023.1175000 (PMC10311032; doi:10.3389/fonc.2023.1175000)
Supplement: Supplementary File S2 — Clinical information of GSE58812. [file DataSheet_2.docx]

id Age MFS fustat OS ER PR HER2

GSM1419942 age at diag: 72.79124 mfs (days): 1184 death: 1 os (days): 1520 er-ihc: 0 pr-ihc: 0 her2-ihc: 0

GSM1419943 age at diag: 66.26421 mfs (days): 984 death: 1 os (days): 1281 er-ihc: 0 pr-ihc: 0 her2-ihc: 0

GSM1419944 age at diag: 67.75633 mfs (days): 877 death: 1 os (days): 1066 er-ihc: 0 pr-ihc: 0 her2-ihc: 0

GSM1419945 age at diag: 49.54963 mfs (days): 905 death: 1 os (days): 1050 er-ihc: 0 pr-ihc: 0 her2-ihc: 0

GSM1419946 age at diag: 84.63245 mfs (days): 340 death: 1 os (days): 422 er-ihc: 0 pr-ihc: 0 her2-ihc: 0

GSM1419947 age at diag: 36.98015 mfs (days): 2081 death: 0 os (days): 2081 er-ihc: 0 pr-ihc: 0 her2-ihc: 0

GSM1419948 age at diag: 59.01985 mfs (days): 4517 death: 0 os (days): 4517 er-ihc: 0 pr-ihc: 0 her2-ihc: 0

GSM1419949 age at diag: 68.18891 mfs (days): 3070 death: 0 os (days): 3070 er-ihc: 0 pr-ihc: 0 her2-ihc: 0

GSM1419950 age at diag: 67.80835 mfs (days): 395 death: 1 os (days): 618 er-ihc: 0 pr-ihc: 0 her2-ihc: 0

GSM1419951 age at diag: 43.55921 mfs (days): 5150 death: 0 os (days): 5150 er-ihc: 0 pr-ihc: 0 her2-ihc: 0

GSM1419952 age at diag: 62.03149 mfs (days): 4996 death: 0 os (days): 4996 er-ihc: 0 pr-ihc: 0 her2-ihc: 0

GSM1419953 age at diag: 65.05681 mfs (days): 419 death: 1 os (days): 480 er-ihc: 0 pr-ihc: 0 her2-ihc: 0

GSM1419954 age at diag: 63.154 mfs (days): 4815 death: 0 os (days): 4815 er-ihc: 0 pr-ihc: 0 her2-ihc: 0

GSM1419955 age at diag: 63.58111 mfs (days): 1879 death: 0 os (days): 1879 er-ihc: 0 pr-ihc: 0 her2-ihc: 0

GSM1419956 age at diag: 73.15811 mfs (days): 1081 death: 1 os (days): 1081 er-ihc: 0 pr-ihc: 0 her2-ihc: 0

GSM1419957 age at diag: 43.04175 mfs (days): 4939 death: 0 os (days): 4939 er-ihc: 0 pr-ihc: 0 her2-ihc: 0

GSM1419958 age at diag: 49.83984 mfs (days): 4783 death: 0 os (days): 4783 er-ihc: 0 pr-ihc: 0 her2-ihc: 0

GSM1419968 age at diag: 54.05613 mfs (days): 4824 death: 0 os (days): 4824 er-ihc: 0 pr-ihc: 0 her2-ihc: 0

GSM1419969 age at diag: 50.11088 mfs (days): 495 death: 1 os (days): 767 er-ihc: 0 pr-ihc: 0 her2-ihc: 0

GSM1419970 age at diag: 57.0924 mfs (days): 4562 death: 0 os (days): 4562 er-ihc: 0 pr-ihc: 0 her2-ihc: 0

GSM1419971 age at diag: 65.5551 mfs (days): 517 death: 1 os (days): 871 er-ihc: 0 pr-ihc: 0 her2-ihc: 0

GSM1419972 age at diag: 40.61876 mfs (days): 4572 death: 0 os (days): 4572 er-ihc: 0 pr-ihc: 0 her2-ihc: 0

GSM1419973 age at diag: 76.37782 mfs (days): 1017 death: 1 os (days): 1055 er-ihc: 0 pr-ihc: 0 her2-ihc: 0

GSM1419974 age at diag: 50.53251 mfs (days): 799 death: 1 os (days): 1430 er-ihc: 0 pr-ihc: 0 her2-ihc: 0

GSM1419975 age at diag: 53.1718 mfs (days): 4270 death: 0 os (days): 4270 er-ihc: 0 pr-ihc: 0 her2-ihc: 0

GSM1419976 age at diag: 78.28063 mfs (days): 3884 death: 0 os (days): 3884 er-ihc: 0 pr-ihc: 0 her2-ihc: 0

GSM1419977 age at diag: 29.60164 mfs (days): 2034 death: 0 os (days): 2034 er-ihc: 0 pr-ihc: 0 her2-ihc: 0

GSM1419978 age at diag: 61.3306 mfs (days): 2593 death: 0 os (days): 2593 er-ihc: 0 pr-ihc: 0 her2-ihc: 0

GSM1419979 age at diag: 74.05339 mfs (days): 3786 death: 0 os (days): 3786 er-ihc: 0 pr-ihc: 0 her2-ihc: 0

GSM1419980 age at diag: 44.13963 mfs (days): 4143 death: 0 os (days): 4143 er-ihc: 0 pr-ihc: 0 her2-ihc: 0

GSM1419981 age at diag: 54.57084 mfs (days): 4147 death: 0 os (days): 4147 er-ihc: 0 pr-ihc: 0 her2-ihc: 0

GSM1419982 age at diag: 53.54689 mfs (days): 3809 death: 0 os (days): 3809 er-ihc: 0 pr-ihc: 0 her2-ihc: 0

GSM1419983 age at diag: 72.74196 mfs (days): 1217 death: 0 os (days): 1217 er-ihc: 0 pr-ihc: 0 her2-ihc: 0

GSM1419984 age at diag: 58.45859 mfs (days): 3393 death: 0 os (days): 3393 er-ihc: 0 pr-ihc: 0 her2-ihc: 0

GSM1419985 age at diag: 71.5373 mfs (days): 616 death: 0 os (days): 616 er-ihc: 0 pr-ihc: 0 her2-ihc: 0

GSM1419986 age at diag: 43.25257 mfs (days): 3492 death: 0 os (days): 3492 er-ihc: 0 pr-ihc: 0 her2-ihc: 0

GSM1419987 age at diag: 62.23409 mfs (days): 3628 death: 0 os (days): 3628 er-ihc: 0 pr-ihc: 0 her2-ihc: 0

GSM1419988 age at diag: 38.85558 mfs (days): 3473 death: 0 os (days): 3473 er-ihc: 0 pr-ihc: 0 her2-ihc: 0

GSM1419989 age at diag: 50.95688 mfs (days): 65 death: 0 os (days): 65 er-ihc: 0 pr-ihc: 0 her2-ihc: 0

GSM1419990 age at diag: 42.02875 mfs (days): 3221 death: 0 os (days): 3221 er-ihc: 0 pr-ihc: 0 her2-ihc: 0

GSM1419991 age at diag: 74.37646 mfs (days): 490 death: 1 os (days): 822 er-ihc: 0 pr-ihc: 0 her2-ihc: 0

GSM1419992 age at diag: 57.34155 mfs (days): 3001 death: 0 os (days): 3001 er-ihc: 0 pr-ihc: 0 her2-ihc: 0

GSM1419993 age at diag: 57.71663 mfs (days): 2125 death: 0 os (days): 2125 er-ihc: 0 pr-ihc: 0 her2-ihc: 0

GSM1419994 age at diag: 76.99384 mfs (days): 163 death: 1 os (days): 163 er-ihc: 0 pr-ihc: 0 her2-ihc: 0

GSM1419995 age at diag: 37.58522 mfs (days): 434 death: 1 os (days): 1047 er-ihc: 0 pr-ihc: 0 her2-ihc: 0

GSM1419996 age at diag: 40.9692 mfs (days): 2887 death: 0 os (days): 2887 er-ihc: 0 pr-ihc: 0 her2-ihc: 0

GSM1419997 age at diag: 31.69062 mfs (days): 2658 death: 0 os (days): 2658 er-ihc: 0 pr-ihc: 0 her2-ihc: 0

GSM1419998 age at diag: 80.21902 mfs (days): 20 death: 0 os (days): 20 er-ihc: 0 pr-ihc: 0 her2-ihc: 0

GSM1419999 age at diag: 73.84805 mfs (days): 2624 death: 0 os (days): 2781 er-ihc: 0 pr-ihc: 0 her2-ihc: 0

GSM1420000 age at diag: 55.96441 mfs (days): 2672 death: 0 os (days): 2672 er-ihc: 0 pr-ihc: 0 her2-ihc: 0

GSM1420001 age at diag: 65.50308 mfs (days): 2569 death: 0 os (days): 2569 er-ihc: 0 pr-ihc: 0 her2-ihc: 0

GSM1420002 age at diag: 58.85831 mfs (days): 1443 death: 1 os (days): 2261 er-ihc: 0 pr-ihc: 0 her2-ihc: 0

GSM1420003 age at diag: 49.14716 mfs (days): 2086 death: 0 os (days): 2086 er-ihc: 0 pr-ihc: 0 her2-ihc: 0

GSM1420004 age at diag: 56.10951 mfs (days): 2527 death: 0 os (days): 2527 er-ihc: 0 pr-ihc: 0 her2-ihc: 0

GSM1420005 age at diag: 82.6694 mfs (days): 811 death: 1 os (days): 811 er-ihc: 0 pr-ihc: 0 her2-ihc: 0

GSM1420006 age at diag: 56.07118 mfs (days): 2293 death: 0 os (days): 2293 er-ihc: 0 pr-ihc: 0 her2-ihc: 0

GSM1420007 age at diag: 52.5284 mfs (days): 2456 death: 0 os (days): 2456 er-ihc: 0 pr-ihc: 0 her2-ihc: 0

GSM1420008 age at diag: 57.3306 mfs (days): 2418 death: 0 os (days): 2418 er-ihc: 0 pr-ihc: 0 her2-ihc: 0

GSM1420009 age at diag: 54.5024 mfs (days): 1950 death: 0 os (days): 1950 er-ihc: 0 pr-ihc: 0 her2-ihc: 0

GSM1420010 age at diag: 60.61054 mfs (days): 1087 death: 1 os (days): 1638 er-ihc: 0 pr-ihc: 0 her2-ihc: 0

GSM1420011 age at diag: 71.72895 mfs (days): 1466 death: 0 os (days): 1466 er-ihc: 0 pr-ihc: 0 her2-ihc: 0

GSM1420012 age at diag: 79.1102 mfs (days): 1083 death: 1 os (days): 1632 er-ihc: 0 pr-ihc: 0 her2-ihc: 0

GSM1420013 age at diag: 43.10472 mfs (days): 2315 death: 0 os (days): 2315 er-ihc: 0 pr-ihc: 0 her2-ihc: 0

GSM1420014 age at diag: 50.12183 mfs (days): 2080 death: 0 os (days): 2080 er-ihc: 0 pr-ihc: 0 her2-ihc: 0

GSM1420015 age at diag: 57.40999 mfs (days): 2009 death: 0 os (days): 2011 er-ihc: 0 pr-ihc: 0 her2-ihc: 0

GSM1420016 age at diag: 59.21424 mfs (days): 2063 death: 0 os (days): 2063 er-ihc: 0 pr-ihc: 0 her2-ihc: 0

GSM1420017 age at diag: 62.63381 mfs (days): 2289 death: 0 os (days): 2289 er-ihc: 0 pr-ihc: 0 her2-ihc: 0

GSM1420018 age at diag: 70.54346 mfs (days): 555 death: 1 os (days): 839 er-ihc: 0 pr-ihc: 0 her2-ihc: 0

GSM1420019 age at diag: 66.00137 mfs (days): 500 death: 1 os (days): 601 er-ihc: 0 pr-ihc: 0 her2-ihc: 0

GSM1420020 age at diag: 67.75085 mfs (days): 1560 death: 0 os (days): 1560 er-ihc: 0 pr-ihc: 0 her2-ihc: 0

GSM1420021 age at diag: 41.40726 mfs (days): 599 death: 1 os (days): 617 er-ihc: 0 pr-ihc: 0 her2-ihc: 0

GSM1420022 age at diag: 36.15332 mfs (days): 3277 death: 0 os (days): 3277 er-ihc: 0 pr-ihc: 0 her2-ihc: 0

GSM1420023 age at diag: 67.32649 mfs (days): 2032 death: 0 os (days): 2032 er-ihc: 0 pr-ihc: 0 her2-ihc: 0

GSM1420024 age at diag: 42.69678 mfs (days): 3341 death: 0 os (days): 3341 er-ihc: 0 pr-ihc: 0 her2-ihc: 0

GSM1420025 age at diag: 37.85079 mfs (days): 3351 death: 0 os (days): 3351 er-ihc: 0 pr-ihc: 0 her2-ihc: 0

GSM1420026 age at diag: 45.37166 mfs (days): 2524 death: 0 os (days): 2524 er-ihc: 0 pr-ihc: 0 her2-ihc: 0

GSM1420027 age at diag: 30.14647 mfs (days): 3053 death: 0 os (days): 3053 er-ihc: 0 pr-ihc: 0 her2-ihc: 0

GSM1420028 age at diag: 50.42026 mfs (days): 1158 death: 1 os (days): 1443 er-ihc: 0 pr-ihc: 0 her2-ihc: 0

GSM1420029 age at diag: 46.32991 mfs (days): 3148 death: 0 os (days): 3148 er-ihc: 0 pr-ihc: 0 her2-ihc: 0

GSM1420030 age at diag: 65.78782 mfs (days): 2991 death: 0 os (days): 2991 er-ihc: 0 pr-ihc: 0 her2-ihc: 0

GSM1420031 age at diag: 58.28337 mfs (days): 1422 death: 0 os (days): 1422 er-ihc: 0 pr-ihc: 0 her2-ihc: 0

GSM1420032 age at diag: 66.36824 mfs (days): 783 death: 0 os (days): 818 er-ihc: 0 pr-ihc: 0 her2-ihc: 0

GSM1420033 age at diag: 68.84052 mfs (days): 2570 death: 0 os (days): 2570 er-ihc: 0 pr-ihc: 0 her2-ihc: 0

GSM1420034 age at diag: 64.41615 mfs (days): 2619 death: 0 os (days): 2619 er-ihc: 0 pr-ihc: 0 her2-ihc: 0

GSM1420035 age at diag: 46.03696 mfs (days): 705 death: 1 os (days): 986 er-ihc: 0 pr-ihc: 0 her2-ihc: 0

GSM1420036 age at diag: 53.75222 mfs (days): 2648 death: 0 os (days): 2648 er-ihc: 0 pr-ihc: 0 her2-ihc: 0

GSM1420037 age at diag: 29.0486 mfs (days): 2717 death: 0 os (days): 2717 er-ihc: 0 pr-ihc: 0 her2-ihc: 0

GSM1420038 age at diag: 57.78234 mfs (days): 940 death: 1 os (days): 1185 er-ihc: 0 pr-ihc: 0 her2-ihc: 0

GSM1420039 age at diag: 66.02053 mfs (days): 527 death: 1 os (days): 619 er-ihc: 0 pr-ihc: 0 her2-ihc: 0

GSM1420040 age at diag: 67.40588 mfs (days): 1632 death: 0 os (days): 1632 er-ihc: 0 pr-ihc: 0 her2-ihc: 0

GSM1420041 age at diag: 70.26421 mfs (days): 925 death: 1 os (days): 1734 er-ihc: 0 pr-ihc: 0 her2-ihc: 0

GSM1420042 age at diag: 52.00547 mfs (days): 2219 death: 0 os (days): 2219 er-ihc: 0 pr-ihc: 0 her2-ihc: 0

GSM1420043 age at diag: 58.67488 mfs (days): 875 death: 1 os (days): 892 er-ihc: 0 pr-ihc: 0 her2-ihc: 0

GSM1420044 age at diag: 55.00342 mfs (days): 1892 death: 0 os (days): 1892 er-ihc: 0 pr-ihc: 0 her2-ihc: 0

GSM1420045 age at diag: 28.48186 mfs (days): 2009 death: 0 os (days): 2009 er-ihc: 0 pr-ihc: 0 her2-ihc: 0

GSM1420046 age at diag: 58.23956 mfs (days): 2025 death: 0 os (days): 2025 er-ihc: 0 pr-ihc: 0 her2-ihc: 0

GSM1420047 age at diag: 54.74059 mfs (days): 1664 death: 0 os (days): 1664 er-ihc: 0 pr-ihc: 0 her2-ihc: 0

GSM1420048 age at diag: 69.86995 mfs (days): 1633 death: 0 os (days): 1633 er-ihc: 0 pr-ihc: 0 her2-ihc: 0

GSM1420049 age at diag: 69.51129 mfs (days): 1780 death: 0 os (days): 1780 er-ihc: 0 pr-ihc: 0 her2-ihc: 0

GSM1420050 age at diag: 44.83231 mfs (days): 1639 death: 0 os (days): 1639 er-ihc: 0 pr-ihc: 0 her2-ihc: 0

GSM1420051 age at diag: 52.32581 mfs (days): 783 death: 1 os (days): 904 er-ihc: 0 pr-ihc: 0 her2-ihc: 0

GSM1420052 age at diag: 49.79329 mfs (days): 1647 death: 0 os (days): 1647 er-ihc: 0 pr-ihc: 0 her2-ihc: 0

GSM1420053 age at diag: 53.14716 mfs (days): 1547 death: 0 os (days): 1547 er-ihc: 0 pr-ihc: 0 her2-ihc: 0

GSM1420054 age at diag: 66.30801 mfs (days): 1262 death: 0 os (days): 1262 er-ihc: 0 pr-ihc: 0 her2-ihc: 0

GSM1420055 age at diag: 43.48255 mfs (days): 1430 death: 0 os (days): 1430 er-ihc: 0 pr-ihc: 0 her2-ihc: 0

GSM1420056 age at diag: 47.58385 mfs (days): 415 death: 0 os (days): 1381 er-ihc: 0 pr-ihc: 0 her2-ihc: 0

GSM1420057 age at diag: 52.41341 mfs (days): 1027 death: 0 os (days): 1390 er-ihc: 0 pr-ihc: 0 her2-ihc: 0
